# TGF-β stimulation in human and murine cells reveals commonly affected biological processes and pathways at transcription level

**DOI:** 10.1186/1752-0509-8-55

**Published:** 2014-05-15

**Authors:** Khalid Abnaof, Nikhil Mallela, Gudrun Walenda, Steffen K Meurer, Kristin Seré, Qiong Lin, Bert Smeets, Kurt Hoffmann, Wolfgang Wagner, Martin Zenke, Ralf Weiskirchen, Holger Fröhlich

**Affiliations:** 1 Bonn-Aachen International Center for IT, University of Bonn, Dahlmannstr. 2, 53113 Bonn, Germany; 2 Helmholtz-Institute for Biomedical Engineering, Stem Cell Biology and Cellular Engineering, RWTH Aachen University, Pauwelsstr. 20, 52074 Aachen, Germany; 3 Institute of Clinical Chemistry and Pathobiochemistry, RWTH Aachen University, Pauwelsstr. 30, 52074 Aachen, Germany; 4 Institute for Molecular Bio-Technology, RWTH Aachen University, Forckenbeckstr. 6, 52074 Aachen, Germany; 5 Genetics and Molecular Cell Biology, CARIM School for Cardiovascular Diseases, Maastricht University, Universiteitssingel 50, 6229 ER Maastricht, Netherlands; 6 Institute for Molecular Biotechnology, Bioanalytical Resource Centre Aachen, RWTH Aachen University, Worringerweg 1, 52056 Aachen, Germany

**Keywords:** TGF-β, Microarray, Time-course analysis, Gene set analysis, Clustering, Functional similarity

## Abstract

**Background:**

The TGF-β signaling pathway is a fundamental pathway in the living cell, which plays a key role in many central cellular processes. The complex and sometimes contradicting mechanisms by which TGF-β yields phenotypic effects are not yet completely understood. In this study we investigated and compared the transcriptional response profile of TGF-β1 stimulation in different cell types. For this purpose, extensive experiments are performed and time-course microarray data are generated in human and mouse parenchymal liver cells, human mesenchymal stromal cells and mouse hematopoietic progenitor cells at different time points. We applied a panel of bioinformatics methods on our data to uncover common patterns in the dynamic gene expression response in respective cells.

**Results:**

Our analysis revealed a quite variable and multifaceted transcriptional response profile of TGF-β1 stimulation, which goes far beyond the well-characterized classical TGF-β1 signaling pathway. Nonetheless, we could identify several commonly affected processes and signaling pathways across cell types and species. In addition our analysis suggested an important role of the transcription factor *EGR1*, which appeared to have a conserved influence across cell-types and species. Validation via an independent dataset on A549 lung adenocarcinoma cells largely confirmed our findings. Network analysis suggested explanations, how TGF-β1 stimulation could lead to the observed effects.

**Conclusions:**

The analysis of dynamical transcriptional response to TGF-β treatment experiments in different human and murine cell systems revealed commonly affected biological processes and pathways, which could be linked to TGF-β1 via network analysis. This helps to gain insights about TGF-β pathway activities in these cell systems and its conserved interactions between the species and tissue types.

## Background

The transforming growth factor-beta1 (TGF-β1) signaling pathway is a fundamental pathway in the living cell, which plays a role in many central cellular processes. The TGF-β superfamily contains over 30 different proteins, such as BMPs, Activins, Inhibins, and the TGF-β1 isoforms [1-3]. The pathway contributes to regulation of various cellular processes, such as apoptosis, cell differentiation, cell growth as well as tumor suppression and immune regulation processes [4].

There are three TGF-β isoforms (TGF-β1, TGF-β2, TGF-β3) which have different physiological and pathological effects on epithelial, endothelial, lymphatic, myeloid and mesenchymal tissues [5]. The TGF-β pathway is one of the most studied pathways [6-10]. However, the complex and sometimes contradicting mechanisms by which TGF-β yields phenotypic effects is not yet completely understood [5]. The classical TGF-β1 pathway is already well established since several years [7]. However, the identification of alternative signaling pathways that contain different receptors and Smad proteins has increased the overall complexity of the TGF-β1 signaling pathway [11]. Additional file 1: Figure S1 shows a simplified cartoon sketch comprising mainly Smads cascades in the TGF-β1 signaling pathway.

In this study we investigated and compared downstream effects of TGF-β1 stimulation on the dynamical response of gene expression in mouse and human in different cell and tissue types. Two types of mouse hematopoietic progenitor cells were used: multipotent progenitor (MPP) and dendritic cell (DC) committed progenitors, referred to as common dendritic progenitor (CDP) cells. CDP differentiate from MPP and give rise to two types of DC: plasmacytoid DC (pDC) and conventional DC (cDC). MPP and CDP were obtained from bone marrow by *in vitro* culture with a specific cytokine cocktail and FACS sorting [12,13]. Furthermore, we employed human mesenchymal stromal cells (MSC), which differentiate into osteocytes, chondrocytes or adipocytes [14-16]. Finally, primary murine hepatocytes (HPC) and immortalized human hepatocytes (human HPC, HepG2) cells were used. We have taken these different cell types for three reasons: (i) All these cells are highly responsive to TGF-β. (ii) The different cell types reflect different degrees of differentiation. (iii) The different cells show a variable response to TGF-β. While in hepatocytes TGF-β induces apoptosis, multipotent progenitors initiate a differentiation programme in response to TGF-β.

Very little and vague information is known about the detailed influence of TGF-β1 in these different cell systems. For example, TGF-β1 is known to be necessary for MSC proliferation. It is essential for chondrogenic differentiation. On the other hand, TGF-β1 participates in inhibition of adipogenic and osteogenic differentiation. Furthermore, there are evidences, that TGF-β1 contributes to supporting myogenic differentiation of MSC [17-19]. There are also evidences that the TGF-β pathway play a role in the induction of cellular senescence in MSC [20]. Although TGF-β1 triggers primary early responses (e.g. Smad activation) and EMT in human HPC (HepG2) cells, cell cycle arrest and apoptosis are generally not promoted by TGF-β1 [21,22]. Furthermore, TGF-β1 is known to be crucial for development of Langerhans cells, the cutaneous contingent of migratory dendritic cells, both *in vivo* and *in vitro* and it evidently contributes in accelerating their differentiation and directing their subsets specification toward cDCs [12,23-25].

We used a panel of bioinformatics methods, ranging from statistical testing over functional and promoter sequence analysis to clustering for pattern discovery in our gene expression time series data. Only one gene, the SKI-like oncogene (*Skil*), was commonly found to be differentially expressed (DE) in all cell types. *Skil* is a component of the SMAD-pathway, which regulates cell growth and differentiation. Moreover, *Smad7* that blocks TGF-β receptor activity seems to play a major common role, because it was identified as DE in most cell types. Despite of the differences on the level of individual genes we observed a conserved effect of TGF-β1 stimulation on a number of biological processes and pathways. Moreover, we could identify a few overrepresented transcription factor binding sites, which were commonly found in several cell types. Specifically EGR1 seems to have major relevance for the transcriptional stimulation response in mouse and human.

By analysis of an independent dataset on human A549 lung adenocarcinoma cells (CRL) from GEO (access No. GSE17708) [26] we were able to reproduce a highly significant proportion of the commonly identified biological processes, pathways and transcriptional factors in our datasets. Network analysis suggests explanations, how TGF-β1 stimulation could lead to the observed effects.

## Results and discussion

### Time series transcriptome measurements

All cell types were treated with TGF-β in three biological replicates. TGF-β treatment concentrations were optimized in each cell type to show a maximal effect. Extracted RNA samples were hybridized to microarrays (Affymetrix Gene 1.0 ST) for genome-wide transcriptome analysis. Mouse progenitor cells and HepG2 cells were measured at 6 successive time points, mouse primary HPC cells at 5, and human MSCs at 4 different time points. Additional file 2: Table S1 gives an overview of our experiments and the measured time-points, the “Methods” section gives details about cell cultures, stimulation, RNA-isolation and array hybridization in our experiments.

### Differential gene expression

#### Transcriptional response is highly tissue specific on gene level

We employed the “betr” method [27] to quantify the probability of differential expression of genes in whole time-courses (see Methods). Using this approach we were able to assess differential gene expression for each gene in each cell type in a comparable manner. We considered a gene to have differential time-course expression (DE), if it had a probability of >99% and was at least two-fold up- or down-regulated at one time point minimum (Additional file 1: Figures S2 a & b, Additional file 2: Tables S2 & S8).

The strongest stimulatory effect of TGF-β1 was observed in CDP cells (614 genes). Eight out of these genes in CDP are already known to play a role in the TGF-β pathway (*Tgfb3, Smad7, Thbs1, Tgfbr1, Smurf1, Smad3, Smad6, Tgfbr2*). In mouse HPC a significantly lower number of DE genes were found compared to other cell types.

We conducted set comparisons of DE genes across cell types. It is worth mentioning in this context that comparisons between mouse and human genes were done on the basis of homologous genes (see Methods). Not surprisingly, the found overlap was particularly high among mouse hematopoietic progenitor cells (MPP and CDP). These were 173 genes, which equals a harmonic mean of above 41% of DE genes in both cell types (Additional file 1: Figure S2 a). Only two of these genes, namely *Smad7* and *Tgfbr1* are known to play a role in the TGF-β pathway. Three genes (*Lox, Pmepa1, Skil*) are found to be DE in all mouse cells (CDP, MPP and HPC). *Pmepa1* (Prostate Transmembrane Protein) is known to interact with *Smad* and suppress the TGF-β pathway [28,29]. Only the protein-coding gene *Skil* (Ski-like-oncogene) that encodes a protein in the SMAD-pathway [30,31] was found to have a DE time-course in all cell types. In addition, the gene *Smad7* was commonly found in all cell types except mouse HPC cells. 18 genes including *ROR1, C10orf10, SMAD7, FSTL3, GADD45B, JUNB, ZFP36, OLFM2, SPTLC3, ID1, LMCD1, SLC38A3, GXYLT2, SKIL, HES1, RASGEF1B, CITED2* and *PDGFA* were DE in all human cells (MSC, HepG2). The heatmaps in Additional file 1: Figure S3 visualize patterns of temporal behavior for particular groups of genes. Here again we see similarity in gene expressions between mouse dendritic cells.

These findings on one hand stress the similarity of the transcriptional response in MPP and CDP, which is not very surprising given the fact that these cells were both derived from bone marrow. On the other hand they highlight that TGF-β1 treatment affects by far not only genes within the canonical TGF-β1 pathway, but leads to a large number of diverse secondary downstream effects, which are only partially overlapping across different cell types. In other words there is a high tissue specificity of the transcriptional TGF-β stimulation response on the level of individual genes.

#### TGF-β1 pathway genes react time-dependant and tissue-specific

**Figure 1:**
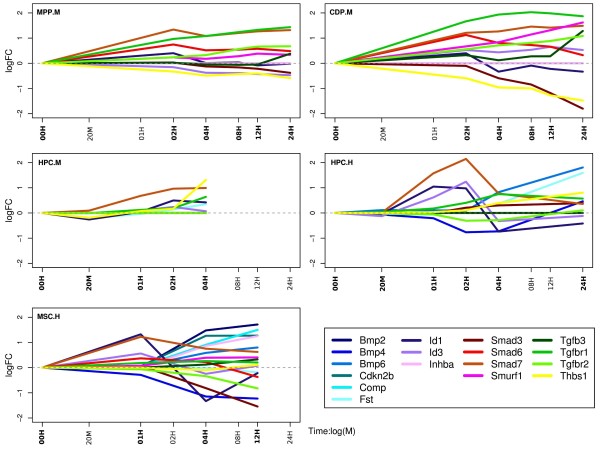
Log2 fold-changes of 17 genes, which are DE in at least one cell type and are known to play a role in the TGF-β pathway (according to KEGG annotation).

We had a closer look at genes, which are known to play a role in the TGF-β1 pathway, such as Bmp(s), Smad(s) and Id(s). In Figure 1 the log2 fold changes of 17 genes involved in the TGF-β1 pathway, which are DE in at least one cell type, are depicted. It can be noticed that almost all genes show time-dependant transcriptional effects. These effects are distinct between early and later time points, with moderate activities until 4 h and mostly higher activities at late times. It can also be noticed that cells of similar origin are more alike. For example, *Bmp2, Bmp4, Bmp6, Cdkn2b* and *Comp* are dys-regulated (i.e. significantly differ from 0 level according to “betr”) only in human and not in mouse tissues. *Fs1* is similar to these genes, but also shows activity in mouse HPC. *Id1* in human cells is up-regulated at earlier time points and a down-regulated after 4 h. *Inhba* shows activity only in MSC cells where its expression after 1 hour constantly increases. *Smad3, Smad6* and *Smad7* reveal similar time courses in mouse MPP and CDP cells and in human MSCs. *Smad3* is increasingly down-regulated over time and the other two genes are always up-regulated. *Smurf1* is always over-expressed and shows a curve that is opposite to *Smad3, Smad6* and *Smad7. Tgfb3* is over-expressed at later time points in MPP and CDP cells and shows almost no activity in the other cell types. *Thbs1* is highly active in all cell types. However, while it is underrepresented in MPP and CDP, it shows elevated expression in mouse and human HPC. *Tgfbr1* and *Tgfbr2* behave similar, in particular in mouse progenitor cells, where *Tgfbr2* is less up-regulated than *Tgfbr1*.

### Time point specific analysis confirms highly tissue specific expression changes on gene expression level

In order to cross-validate our previous analysis, which considers time series as a whole, we conducted also a time-point specific analysis of differential gene expression using linear models for microarray data (Limma). For this purpose we compared the gene expression at 4 hours after stimulation to the initial expression at time point 0. The time period of 4 hours was chosen because at least short-time relevant effects are expected in all cell types after this period.

In the context of this time point specific analysis of transcriptional effects we considered a gene to be differentially expressed (DE) in a given cell type, if FDRBH < 0.01 and the absolute fold changes was (logFC) > 1. The overlap analysis of DE genes at 4 h agrees with the time-course analysis. There are no or very few genes in common between the different cell types except in the case of mouse dendritic cells (Additional file 1: Figure S5 A). Moreover, the direction of regulation (up or down) differs between cell types (Additional file 2: Table S9).

The heatmap in Additional file 1: Figure S4 depicts the log fold changes of all genes, which are DE in at least one cell type. The plot indicates two gene sets, which clearly show a similar behavior in mouse MPP and CDP cell types. The first set contains 36 genes that are over-expressed. The other set (42 genes) is under-expressed. Interestingly, the 36 genes being up-regulated in MPP and CDP cells are not regulated by TGF-β1 in other cell types. Although not DE genes in every cell type, the genes *Smad7, Pmepa1* (beside the gene *Skil*) seem to be up-regulated in all the cells. The rest of the genes are regulated in a rather cell-type specific manner.

### Cluster analysis reveals functionally similar gene groups in different cell types

**Figure 2:**
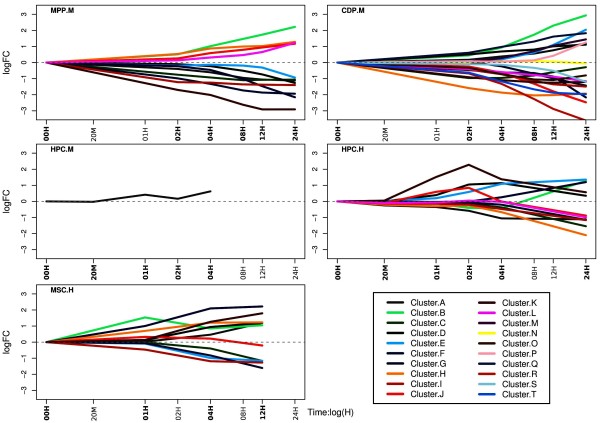
**Cluster mean-curves of log2 fold changes for the different cell types.** For mouse HPC no clusters could be identified and hence all DE genes treated as one group.

We conducted a time series cluster analyses in order to find groups of DE genes showing similar expression changes over time (observed as within cell-type temporal behavior shown in Additional file 1: Figures S3, between cell-types similarities shown in Additional file 1: Figure S4). The cluster analyses yielded 12 clusters in MPP and mouse HPC, 20 in CDP and 11 in human MSC (Additional file 2: Table S3). Genes contained in individual clusters can be found in Additional file 2: Table S14. Figure 2 depicts the mean curves for each of these clusters in each cell type. We investigated the functional similarity of genes across different clusters. For this purpose the R-Package “GOSemSim” [32] utilizing the semantic similarity measure proposed by Wang et al. [33] was employed. Semantic similarities are a means to compare GO annotations of gene pairs in a quantitative manner, for example on the basis of the information content of GO terms. We refer the reader to [34] for an excellent overview.

**Figure 3:**
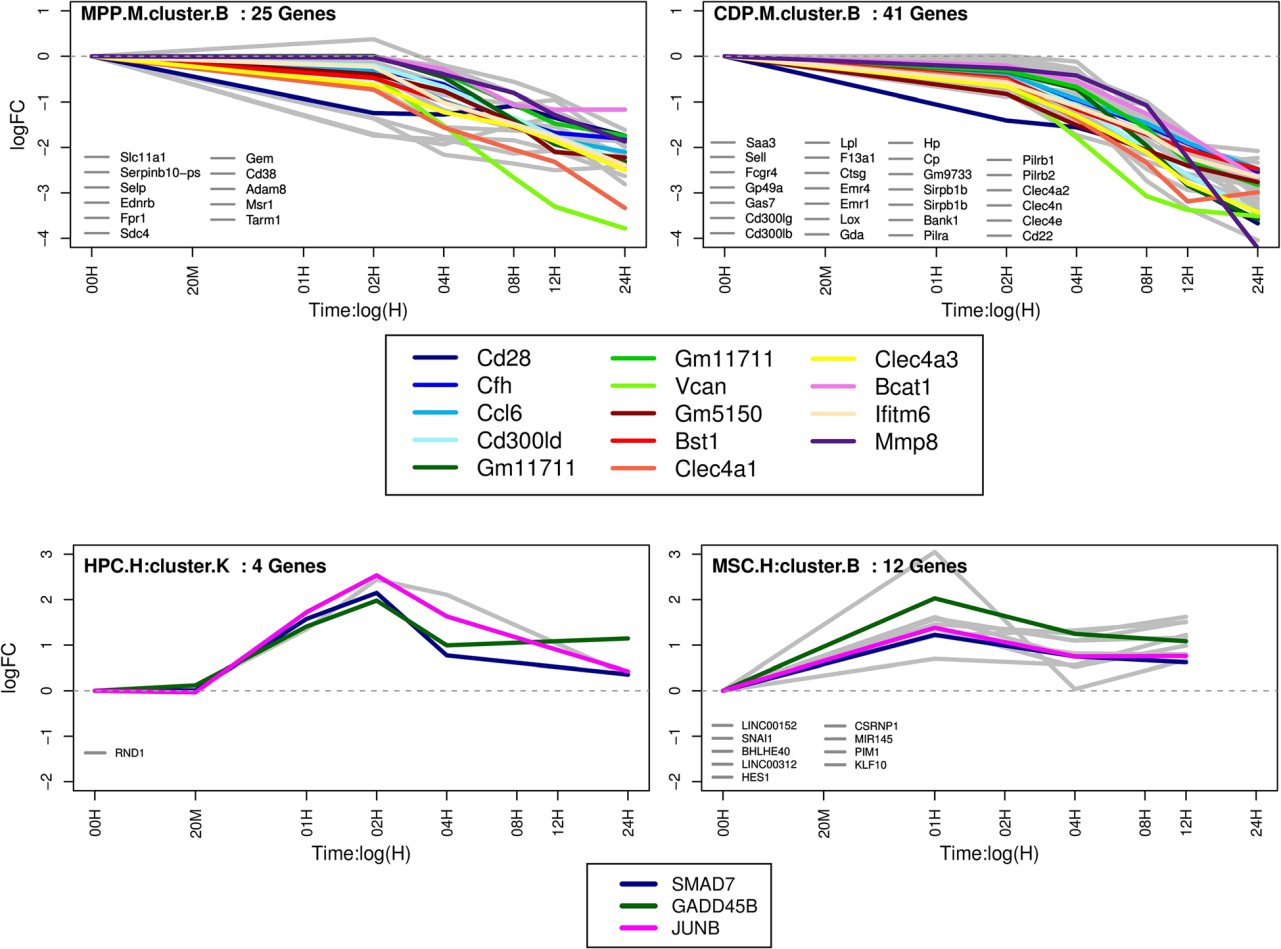
**Log2 fold changes of two groups of functionally similar clusters detected in different cell types.** Genes appearing in more than one cluster are depicted in color, gray curves are cluster-specific genes. Upper group: two similar clusters MPP and CDP. Lower group: two similar clusters in human HPC and MSC.

A heatmap depicting these GO semantic similarities suggested a high functional similarity of genes in several clusters from different cell types (Additional file 1: Figure S6 Additional file 2: Tables S15, S16). In particular cluster B (MPP), and cluster B (CDP) are highly similar to each other (semantic similarity > 0.7). Time-course log2 fold changes of the corresponding genes are shown in (Figure 3 top). As can be noticed the clusters are of different size, but have several genes in common (13 genes). Functional analysis revealed that genes in these clusters are enriched for *cell adhesion molecules (CAMs), valine, leucine and isoleucine biosynthesis, Pantothenate and CoA biosynthesis* and *regulation of cellular extravasation*. Enrichment analysis was conducted here *via* the R-package GOstats [35], which employs a hypergeometric test taking into account the dependency structure among GO terms.

The second group of functionally similar clusters (Figure 3, bottom) contains cluster K (human HPC) and cluster B (MSCs). Genes in these clusters play (among others) a role in *TGF-β* and *Notch signaling* pathways (Additional file 2: Tables S15, S16).

Taken together our cluster analyses showed that despite evident differences on the level of individual genes, functionally similar clusters of genes can be identified across cell types.

### Enrichment analysis reveals commonly affected biological processes, pathways and transcription factors in all cell types

Motivated by our previous findings we asked, whether there were common functional patterns across all cell types. For this purpose we scanned GO terms and KEGG pathways for significant association with differential time course gene expression in each cell type (Additional file 2: Tables S4, S10, S11).

**Figure 4:**
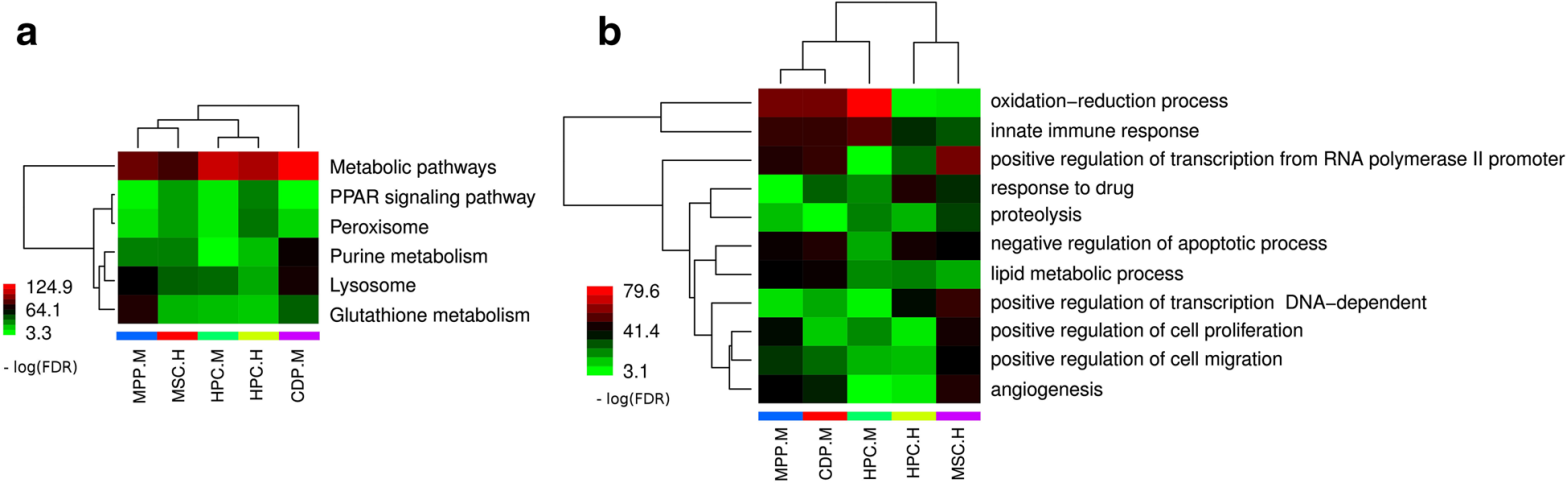
**Clustered heatmaps of (a) the 6 common KEGG pathways and (b) 11 GO terms in different cell types.** The color code indicates the degree of association (−log(FDR)) of a KEGG pathway and GO term to each cell type, respectively.

Our analysis brought up 6 KEGG pathways and 11 GO terms, which were significantly associated to all cell types (FDR < 5%, Figure 4). The 6 KEGG pathways associated to all cell types were: *Metabolic pathways, Glutathione metabolism, Lysosome, Purine metabolism, Peroxisome* and *PPAR signaling pathway*. The 11 GO terms associated to all cells were: *oxidation-reduction process, innate immune response, positive regulation of transcription from RNA polymerase II promoter, negative regulation of apoptotic process, angiogenesis, lipid metabolic process, positive regulation of cell proliferation, positive regulation of cell migration, proteolysis, positive regulation of transcription DNA-dependent* and *response to drug*. The role of TGF-β in apoptosis, cell proliferation as well as immune response is well known. Moreover, an effect of TGF-β perturbation on PPAR signaling has been described in skin fibroblasts [36]. In [37] the authors describe TGF-β mediated oxidative stress and decreased glutathione concentration in fibrosis models. Finally, there is evidence that TGF-β has an effect on angiogensis and cell migration [38]. Hence, our findings largely fit to the current biological knowledge about TGF-β.

### Conserved role of EGR1/2 transcription factors

We analyzed DE genes with respect to overrepresented sequence motifs in their promoter regions with the XXmotif tool [39]. Significant motifs were then compared to known position weight matrices (TRANSFAC) of transcription factors (TFs) *via* STAMP [40].

**Figure 5:**
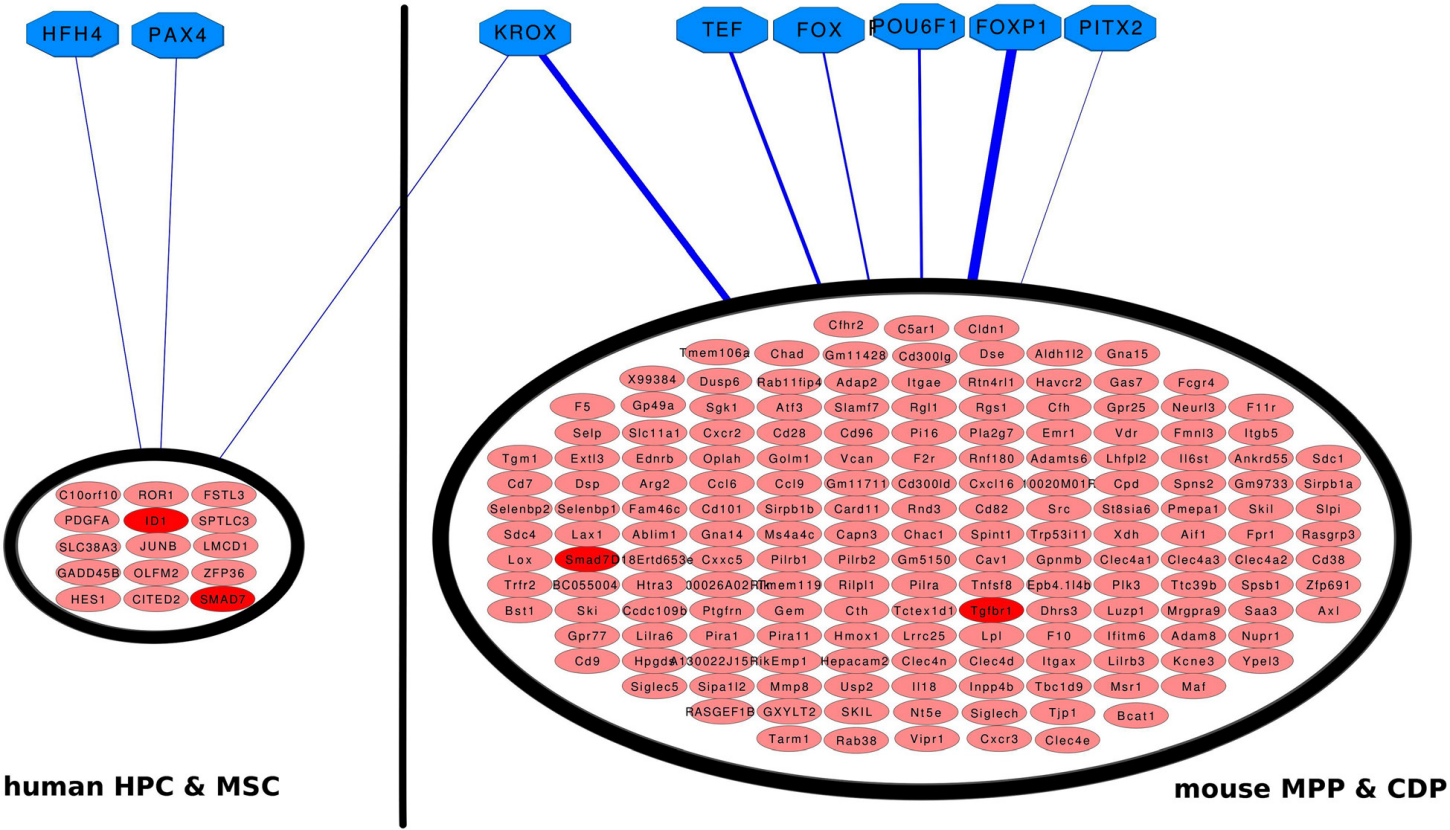
**Network of eight overrepresented TFBS and DE genes containing these binding sites.** For the sake of better visualization only the set of genes being DE in both, HPC and MSC as well as both, MPP and CDP, are shown. Red genes are known to play role in the TGF-β pathway. The width of the blue lines is chosen to be proportional to the average –log E-value, which resulted from the XXmotif analysis.

The analysis in each cell type predicted between 11 and 21 regulating TFBS in the time-course analysis (Additional file 2: Tables S6, S17, S18), except for mouse HPC, where no overrepresented TFBS could be detected. This may be attributed to the small number of 16 DE genes in this cell type. Overlaps were particularly high within mouse MPP and CDP and within human cells.FOXP1, KROX, TEF, POU6F1, FOX and PITX binding sites were commonly identified in mouse MPP and CDP. KROX, HFH4 and PAX4 were found in all human cells. FOX, FOXP1, KROX and TEF were found to be themselves representatives of DE genes. Figure 5 shows a network representation of all eight TFBS together with the set of DE genes containing respective binding sites. The plot reveals a relative clear difference between mouse and human cells with the exception of the KROX TFBS, which appears in all four cell types. KROX represents EGR1 and EGR2.

### Network analysis suggests possible signal transduction pathways in mouse and human

**Figure 6:**
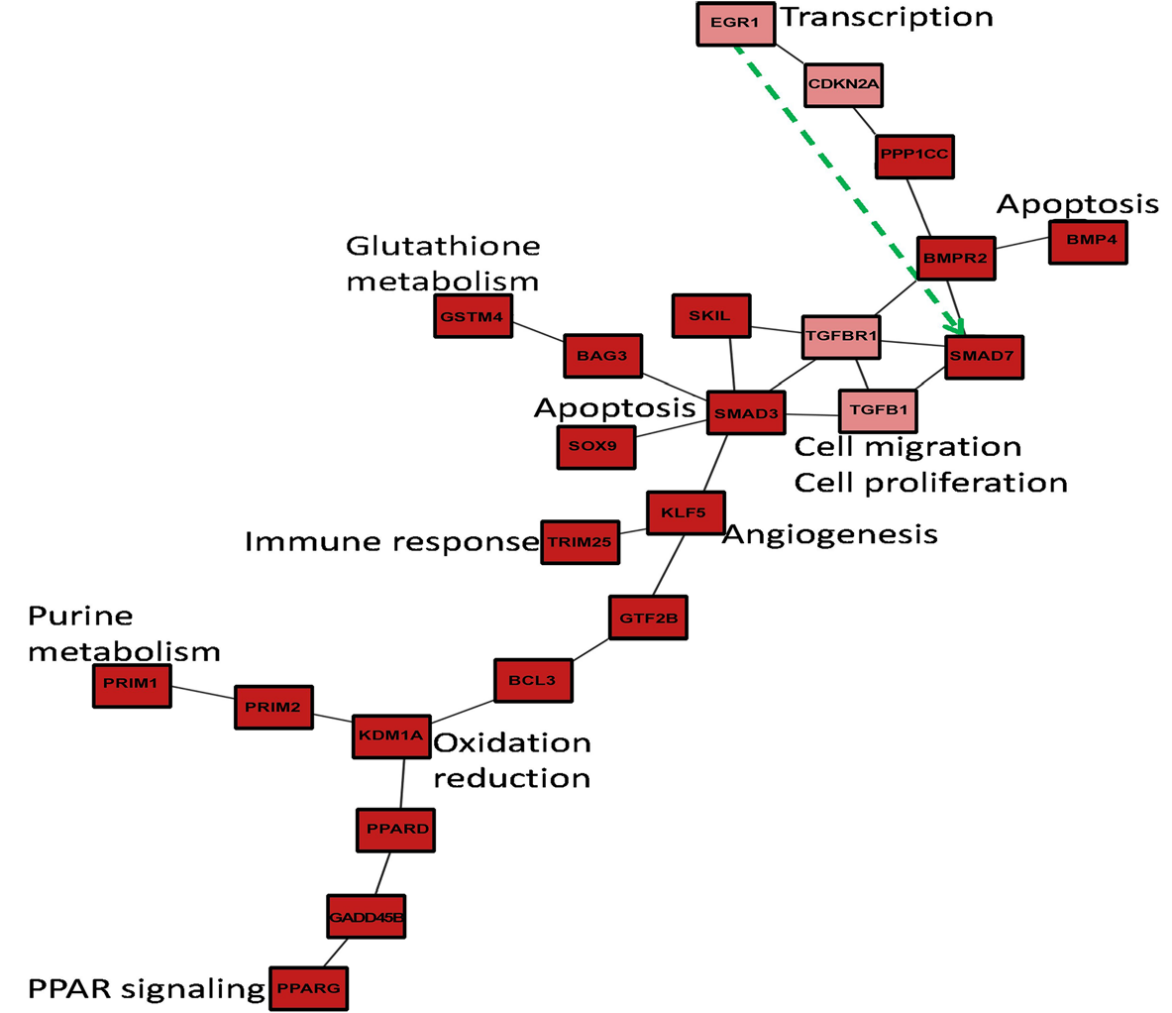
**Human protein-protein interaction network connecting TGFB1, TGFBR1, SMAD7, SKIL, EGR1, PPARG with genes involved into commonly identified biological processes and pathways.** The dashed green line indicates the putative transcriptional regulation of SMAD7 by transcription factor EGR1. The darker the red color of a node the higher the average probability for differential time course expression.

**Figure 7:**
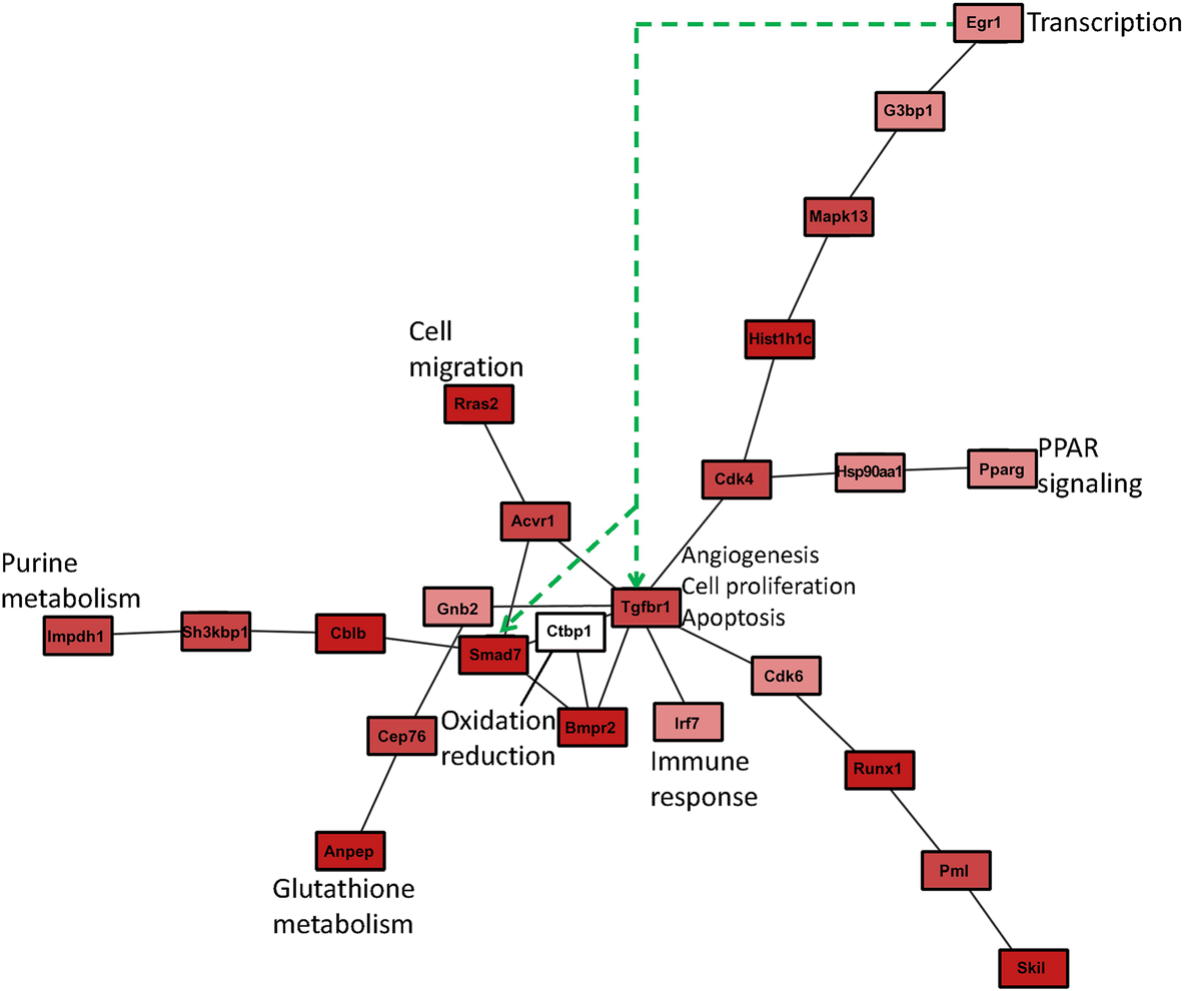
**Murine protein-protein interaction network connecting Tgbfr1, Smad7, Skil, Egr1, Pparg with genes involved into commonly identified biological processes and pathways.** The dashed green line indicates the putative transcriptional regulation of Smad7 and Tgfbr1 by transcription factor Egr1. The darker the red color of a node the higher the average probability for differential time course expression.

In order to better understand, how TGF-β may influence the commonly identified transcription factor, biological processes and the PPAR-pathway we conducted a network analysis. Using protein-protein interaction information from the BioGRID database [41] we reconstructed a mouse and a human specific network depicting dys-regulated paths from TGF-β to *SKIL*, *SMAD7*, *EGR1* as well as genes involved into g*lutathione metabolism, purine metabolism, PPAR signaling*, *oxidation-reduction process, innate immune response, negative regulation of apoptotic process, angiogenesis, positive regulation of cell proliferation* and *positive regulation of cell migration* (Figures 6 and 7; see further details in Methods part)*.*

Our network analysis suggests pathways, by which TGF-β stimulation is possibly propagated via protein-protein interactions to our commonly identified biological processes. Due to the organism specificity of interactome information these pathways show certain differences: Far less protein-protein interactions are known in mouse than in human. In human, for example, negative regulation of apoptosis might be mediated via SMAD3 and SOX9 [42]. In contrast, the GO and network analysis in mouse suggests a direct role of TGFBR1.

### Enrichment of biological processes, pathways and tfbss is reproducible on independent dataset

**Figure 8:**
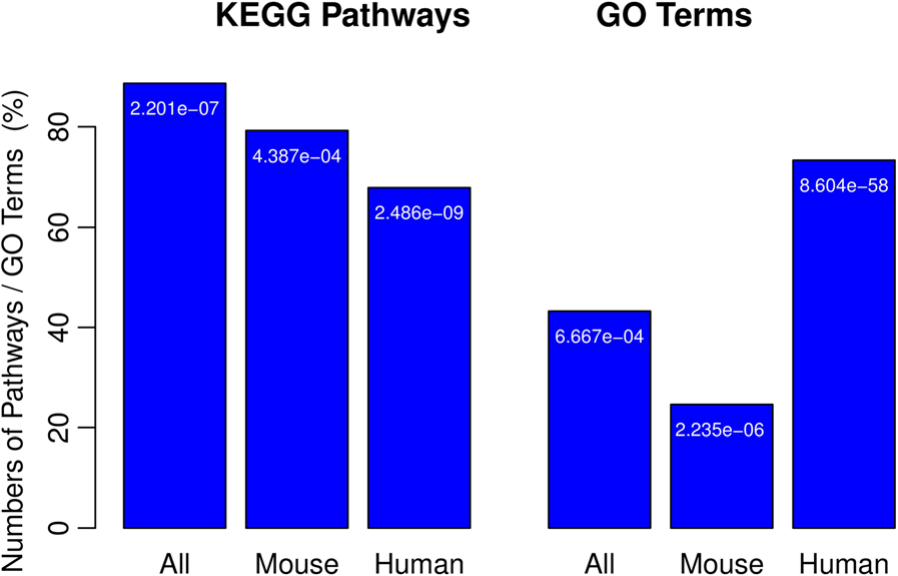
**Percentages of KEGG pathways (left) and GO terms (right) enriched commonly in our cell types that could be reproducibly identified in GSE17708.** The numbers in tip of the bars are the p-value for the null-hypothesis to see the corresponding overlap just by chance (hyper-geometric test).

To validate the central finding from our data, namely the existence of commonly affected biological processes, pathways and transcription factors in all cell types, we downloaded an independent dataset comprising gene expression data measured at 9 time points (0, 0.5, 1, 2, 4, 8, 16, 24, 72 h) after TGF-β stimulation in human A549 lung adenocarcinoma cell-lines (CRL, GSE17708). The dataset was analyzed in the same manner as described for our data before. High fractions of the 11 GO terms and 6 KEGG pathways commonly identified in all of our cell types were also found in GSE17708 (Figure 8, Additional file 2: Table S10). Out of the KEGG pathways and GO terms associated to all of our human cells 70% and 74%, respectively could be reproduced on the independent dataset (Figure 8, Additional file 2: Table S11). Notably, 11 (61%) out of the 18 genes which exhibiting differential time courses in both our human MSC and HPC cells were found also to have differential time-courses in GSE17708 cells, these were *ROR1, SMAD7, FSTL3, GADD45B, JUNB, ZFP36, ID1, LMCD1, GXYLT2, SKIL* and *HES1*. This corresponding fraction is significantly larger than expected by chance (p < 1E-9, hyper-geometric test).

The KROX TFBS (corresponding to TFs EGR1 and EGR2), which was enriched in all of our cell types, was also found in GSE17708. Moreover, the other two TFBS that we identified in our human cells (HFH4, PAX4) were also enriched in the A549 lung cancer cell line (Additional file 2: Tables S17).

Taken together this analysis reveals a high reproducibility of our commonly identified biological processes, pathways as well as TFs.

## Conclusions

We have conducted an in-depth comparison of the dynamical TGF-β1 response profile on gene expression level across several cell types. Despite of a generally high degree of cell type specificity, there appears to be a common functional response, which is conserved across cell types and species (i.e. mouse and human). Our analysis suggests a common effect of TGF-β1 stimulation on apoptosis, cell proliferation, immune response, angiogenesis, cell migration, PPAR signaling, oxidative stress as well as purine and glutathione metabolism. Network analysis gives hints to possible pathways, by which these effects could be mediated.

On the level of individual genes the *SKI*-like oncogene and *Smad7* were differentially expressed in most (*Smad7*) or all (*SKI*-like oncogene) cell types and thus appear to play a major role. *Smad7* is involved into the canonical TGF-β pathway [43]. It is a general antagonist of the TGF-β family (for review see [44]). The *SKI*-like oncogene is a direct target gene of *Smad2*, which regulates its transcription [45]. It plays a role in cell growth and differentiation.

Notably, a high fraction of the biological processes, pathways and TFBS that we identified to be enriched in all our cell types was found also in an independent dataset from a lung cancer cell line. This strengthens the confidence into our results.

In summary our findings indicate that despite a high variability of transcriptional response across cell types and organisms there appears to be a set of commonly affected processes and pathways. In addition, the TFBS analysis suggested a major role of the transcription factor EGR1 in the TGF-β response in human and mouse. Indeed the induction of EGR1 *via* TGF-β stimulation has been already reported earlier [46] and thus fits to the existing knowledge about TGF-β1 − induced transcriptional response in other cell systems.

Previous studies of TGF-β stimulation were mainly limited to one specific cell type (e.g. fibroblasts). In this paper we went beyond this point and conducted experiments in different cell types under as much as possible comparable conditions. In consequence we were able to compare transcriptional responses across cell types and organisms, which revealed common patterns. The identification of common and specific signal transduction pathways that are affected by TGF-β in human and mice will allow us to define potential therapeutic targets and will further enable us to characterize gene expression patterns and complex regulatory networks. In addition, future work using our and other transcriptome data can, for example, address the identification of TGF-β dependent mesenchymal or epithelial gene signatures or the definition of cell specific cancer signatures.

## Methods

### Ethics statement

Animal experiments required for obtaining murine MPP, CDP and HPC were approved by local authorities (Landesamt für Natur, Umwelt und Verbraucherschutz Nordrhein-Westfalen - LANUV NRW) in compliance with the German animal protection law. Experiments were performed at the Institute for Animal Research of the RWTH Aachen University Hospital, under projects 10534A4 and 10718A4, entitled “Untersuchungen zur Hämatopoese aus adulten Blutstammzellen”. Animal maintenance, handling, and anesthesia were performed according to the Federation for Laboratory Animal Science Associations FELASA recommendations.

All samples of human MSC cells were used after patient’s written consent using guidelines approved by the Ethic Committee of the RWTH University of Aachen (Permit number: EK128/09).

### Mouse hematopoietic progenitor cells (MPP & CDP)

#### Cell culture

MPP and CDP were obtained from mouse bone marrow, using *in vitro* culture with a specific cytokine cocktail and FACS sorting [12,13].

### TGF-β1 stimulation

After sorting, MPP and CDP were treated with 10 ng/mL recombinant human TGF-β1 (R&D Systems, Minneapolis, USA) for 2, 4, 8, 12 and 24 h as described in [12] or left untreated. Cells were lysed in 350 μl TRI-Reagent and stored at −80°C.

### RNA isolation

RNA was isolated using the MagMAX-96 for Microarrays kit (Life Technologies, Darmstadt, Germany) according to manufacturer’s protocol.

### Primary mouse hepatocytes and human HepG2 cells (HPC)

Hepatocytes (HPC) represent the most prominent cell population in the liver. Primary HPC are sensitive to TGF-β1, and express the corresponding type I (ALK5), type II (TβRII), and type III (betaglycan) receptors. TGF-β1 promotes cell cycle arrest and apoptosis of primary HPC. In addition, *in vitro* TGF-β1 provokes epithelial-to-mesenchymal transition (EMT)-like processes in this hepatic cell subpopulation, which most likely do not occur *in vivo*[47].

HepG2 cells originate from a 15 year old child with primary hepatoblastoma [48]. They do secrete the major plasma proteins but do not express the hepatitis B virus surface antigen (HBsAg) [49].

### Cell culture

Primary murine HPC were isolated from male C57BL/6 mice according to the collagenase method of Seglen [50]. Cells were plated in collagen coated 6-well dishes at a density of 1.2 × 10^6^ cells using HepatoZYME-SFM (Gibco, Life Technology, Darmstadt, Germany). Four hours after seeding the medium was renewed and cells were grown for a further 24 hrs culture period.

HepG2 (DSMZ: DSM ACC180) were cultured in RPMI (PAA, Pasching, Austria) containing 10% fetal calf serum (PAA), 1 × Penicillin/Streptomycin (Lonza, Cologne, Germany). Medium was renewed every second day. For the experiment, cells were passaged and plated in 6-well dishes using accutase (PAA) at a density of 4 × 10^5^ cells. One day before the experiment, cells were washed with PBS (1×), medium changed to HepatoZYME-SFM (Gibco) and cultured for further 24 hrs.

### TGF-β1 stimulation

One hour before the experiment, the medium was exchanged and cells stimulated with 1 ng/mL recombinant human TGF-β1 (R&D Systems, Minneapolis, USA) for indicated time intervals (HepG2: 0 min, 20 min, 1 h, 2 h, 4 h, 24 h; murine HPC: 0 min, 1 h, 2 h, 4 h). The cells were harvested using Qiazol for cell lysis (Qiagen, Hilden, Germany), directly frozen and stored at −80°C.

### RNA isolation

RNA was isolated using the RNeasy Kit system (Qiagen), performing a DNAse digestion according to the manufacturer’s protocol.

### Human Mesenchymal Stromal Cells (MSC)

Mesenchymal stromal cells (MSC) are found in all supportive tissue as in fat tissue, bone marrow and cord. MSC are characterized by their plastic adherence and their differentiation potential towards adipogenic, osteogenic and chondrogenic lineages. All MSC express the surface markers CD29, CD73, CD90 and CD105 and they lack the expression of CD14, CD31, CD34 and CD45 [14,51].

### Isolation and expansion

MSCs were isolated from mononuclear cells (MNCs) by plastic adherence as described before [16,52,53]. In brief, bone fragments from the caput femoris of patients undergoing femoral head prosthesis were flushed with phosphate- buffered saline (PBS) and washed twice with PBS. MNC were then resuspended in culture medium and seeded into tissue culture flasks. Cells were cultured at 37°C in a humidified atmosphere with 5% CO_2_. The first medium exchange was performed after 48 h to remove nonadherent cells. Thereafter, media changes were performed twice per week and MSCs were passaged when reaching 80-90% of confluence.

### TGF-β1 stimulation

MSC from three different donors were used in an early passage (p3-5) for stimulation with TGF-b1. 1x10^6^ MSC were seeded into 6-well culture plates. When the cells were attached after 24 h 1 ng/mL recombinant TGF-β1 (R&D Systems) was added to the culture media at different time points. The cells were harvested at the same time point with Qiazol (Qiagen) and directly frozen and stored at −80°C.

### RNA isolation

RNA was isolated *via* phenol/chloroform extraction using the miRNeasy Kit (Qiagen), performing a DNAse digestion.

### Genechip hybridization

Human samples were assayed using Affymetrix GeneChip® type “Human Gene 1.0 ST Array” with 34,760 probe-sets and mouse samples were assayed in Affymetrix GeneChip type “Mouse Gene 1.0 ST Array” with 32,321 probe-sets. Hybridization, wash and staining were done according to manufacturer’s recommended standard techniques.

### Normalization and preprocessing

Raw probe intensities were normalized and summarized to expression levels using the FARMS algorithm which utilizes a factor analysis approach [54]. A rigorous quality assessment confirmed a fairly good quality of the chips with exception of mouse HPC chips where Initial chip quality assessment revealed a strong batch effect and one bad chip (replicate no. 1 at time 1 hour). The bad chip was excluded and batch adjustment was performed to alleviate that effect on those chips *via* the “ComBat” method [55].

Affymetrix probe IDs were mapped to Entrez gene IDs using the Bioconductor annotation packages “mogene10sttranscriptcluster.db” in mouse chips and “hugene10sttranscriptcluster.db” in human chips [56,57].

### Differential gene expression

#### Time point specific analysis

Differential gene expression analyses *via* “Limma” Linear Models for Microarray Data [58] using empirical Bayes method [59] was performed by comparing samples at each time point after TGF-β stimulation to the unstimulated cells at time point 0. Statistical dependencies of samples between time points and replicates were considered via a factorial design matrix in “Limma” using a “time” and a “replicate” factor, and contrasts are considered for interaction effects. Corrections for multiple testing was done using the Benjamini & Hochberg’s method [60]. Significant differentially expressed genes are considered those with FDRBH < 0.01 and absolute log2 fold change value (logFC) ≥ 1.

### Analysis of whole time courses

The small number of replicates in our experiments limits the power of statistical testing procedures for assessing differential gene expression at individual time points. Furthermore, the number of measured time points is not the same for each cell type, which complicates any further meta-analysis. Therefore, we employed the “betr” method to analyze whole time series at once [27]. The algorithm of this method uses a random-effects model together with the empirical Bayes method to estimate probabilities for differential expression of whole time courses. Genes were considered to be significant at a probability cutoff of > 99% for the whole time-course analysis and absolute log2 fold change (logFC) ≥ 1. Since “betr” requires the same number of replicates per time point and one chip in mouse HPC had to be omitted due to low quality (see Normalization & Preprocessing) unfortunately in this particular cell line we had to exclude time point 1 h completely from the time-course analysis.

### Cluster analysis

Clustering of gene expression time series was done via the MFDA method proposed in [61]. The method assumes gene expression time series within each given cluster to follow a mean curve plus some Gaussian noise. It decides cluster allocation *via* a Gaussian Mixture Model utilizing an EM algorithm for parameter estimation and decides the optimal number of clusters *via* the Bayesian Information Criterion (BIC).

It is worth mentioning that we applied MFDA not on raw gene expression data here, but on log fold-changes relative to time point 0. The reason was that we wanted to group genes not on the basis of their absolute expression values, but on the basis of similar response to the stimulus.

### KEGG and go analysis

Analyses of pathways in KEGG [43] and biological processes in Gene Ontology project (GO) [62] were performed as follows: The –log(p-value) of all genes in the individual time point analysis and –log(1 – probabilities) of all genes in the whole time-course analysis, respectively, were taken as a ranking score for each transcript. Gene sets of KEGG pathways and GO terms were then tested for their association with these ranking scores *via* a univariate logistic regression based test [63,64]. Resulting p-values of KEGG pathways and GO terms were adjusted according to Benjamini & Yekutieli’s false discovery rate control under dependency [65], and significant KEGG pathways and GO terms reported at a FDR_BY_ cutoff value of 5%.

### Transcription factor binding sites analysis

Analysis of transcription factors binding sites (TFBSs) was performed using the *de novo* sequence motif detection method XXmotifs [39]. Identified sequence motifs were then aligned to known TRANSFAC TFBS via STAMP [66] and the top match is considered. The XXmotif method uses BLAST [67] all-against-all comparisons to mask regions of local homology in order to avoid false positives. The method then performs an enrichment analysis after transforming the found patterns to position weight matrices (PWMs). The STAMP method utilizes a global or un-gapped local alignment to detect DNA motifs similarities to defined PWMs. Furthermore, it considers familial binding profiles, thus improving transcription factors (TF) classification accuracy. TFBSs analysis was done using these methods in each cell type for those genes, which according to the time-course analysis showed a probability of > 0.99% for differential expression. Promoter sequences of the genes under consideration (2Kbp upstream of transcription start site) were obtained from the Ensembl database [68]*via* “biomaRt” [69,70]. Only the top matching motif for each TRANSFAC TFBS was considered and significant TFBSs were reported at an E-Value threshold of 1e-3.

Mapping of TFBS to individual transcription factors was performed via manual inspection of TRANSFAC PWMs. We obtained all proteins which had been used to construct each of the PWMs. With the help of the commercial software GeneGo Metacore® [71] we then mapped protein names to Entrez gene IDs. As a consequence we found for the TFBS *FOXP1* the differentially expressed genes *Foxp2* and *FoxP1* (MPP, CDP mouse cells). For the TFBS *FOX* the human gene *FAU* was identified (human HPC). For *KROX* we found *Egr1/EGR1* (mouse HPC, human MSC) and *EGR2* (human MSC). For *TEF* we identified *Klf3/KLF3* (MPPs, CDPs, and human HPC), *TRIM37* and *USP7* (human HPC, MSC).

### Identification of homologous genes

Human homologs of mouse genes were identified via the KEGG Sequence Similarity Data Base (SSDB), which contains local alignments of amino acid sequences for protein coding genes from different species. We here considered two genes to be homologs, if the alignment E-value was below 1e-30 (bit-score > 112). In case of more than one homologous gene, all are considered.

### Network analysis

Information about protein-protein interactions was collected separately for human and mouse from the BioGRID database version 3.2.109 [41]. Correspondingly, a network comprising 16,011 nodes and 140,471 physical interactions was constructed for human. For mouse the network consisted of 6,233 nodes and 16,100 physical interactions. Nodes in these networks were weighted by the average probability (mean over all cell types from the same organism) for differential time course expression according to the betr model (see above). A “distance” for each edge was then calculated as 2 minus the sum of its incident nodes’ weights. Hence: the smaller the distance the higher the weight of its incident nodes. We used Dijkstra’s algorithm to search for minimum distance (i.e. maximum node weight) path connecting TGFB1 with each of SKIL, SMAD7, EGR1, PPARG and all genes annotated to g*lutathione metabolism, purine metabolism, oxidation-reduction process, innate immune response, negative regulation of apoptotic process, angiogenesis, positive regulation of cell proliferation* and *positive regulation of cell migration*. For each of the last mentioned terms we only kept those genes as representatives which showed the minimum distance to TGFB1. If there were several paths of the same minimum distance, all of them were considered.

In the network for mouse Tgfb1 was not identified and hence we started our analysis with *Tgfbr1* instead.

### Availability of supporting data

The datasets supporting the results of this article are included within the article and its supplemental material files.

In addition, our microarray datasets for the different cell types analyzed here are deposited in the Gene Expression Omnibus database [72] under accession numbers GSE45942 (murine HPC), GSE45945 (human HPC), GSE46019 (human MSC) and GSE46109 (mouse MPP and CDP).

## Abbreviations

TGF-β1: Transforming growth factor-beta; CDP: Common dendritic progenitor(s); HPC: Hepatocytes; Limma: Linear models for microarray data; MPP: Multi-potent progenitor(s); MSC: Mesenchymal stromal cell(s); pDC: Plasmacytoid dendritic cell; cDC: Conventional dendritic cell; A549-CRL: Lung adenocarcinoma cell-line; betr: Bayesian estimation of temporal regulation; limma: Linear models for microarray data; TFBS: Transcription factor binding site; GO: Gene ontology; KEGG: Kyoto encyclopedia of genes and genomes.

## Competing interests

The authors declare that they have no competing interests.

## Authors’ contributions

HF, MZ, RW, KH, BS and KA conceived, designed and coordinated the study. GW, SM, KS, QL, BS and KF prepared the samples and carried out microarray experiments. KA, NM and HF performed data analysis. KA, HF, MZ, RW helped to interpret the results and to draft the manuscript. All authors read and approved the final manuscript.

## Supplementary Material

Additional file 1: Figure S1-S6 Supplemental Material: Tgf- Stimulation in human and murine cells reveals commonly affected biological processes and pathways at transcription level. Click here for file

Additional file 2: Table S1-S18 Tables and excel files with results: Tgf- Stimulation in human and murine cells reveals commonly affected biological processes and pathways at transcription level. Click here for file
