# Supplementary material for: TGF-β stimulation in human and murine cells reveals commonly affected biological processes and pathways at transcription level
Source: BMC Syst Biol. 2014 May 15;8:55. doi: 10.1186/1752-0509-8-55 (PMC4049504; doi:10.1186/1752-0509-8-55)
Supplement: Additional file 1: Figure S1-S6 — Supplemental Material: Tgf- Stimulation in human and murine cells reveals commonly affected biological processes and pathways at transcription level. [file 1752-0509-8-55-S1.docx]

**Supplemental Material: TGF-β Stimulation** **in Human and Murine Cells Reveals Commonly Affected Biological Processes and Pathways at Transcription Level**

Khalid Abnaof, Nikhil Mallela, Gudrun Walenda, Steffen Meurer, Kristin Seré, Qiong Lin, Bert Smeets, Kurt Hoffmann, Wolfgang Wagner, Martin Zenke, Ralf Weiskirchen, Holger Fröhlich

**Figures:**


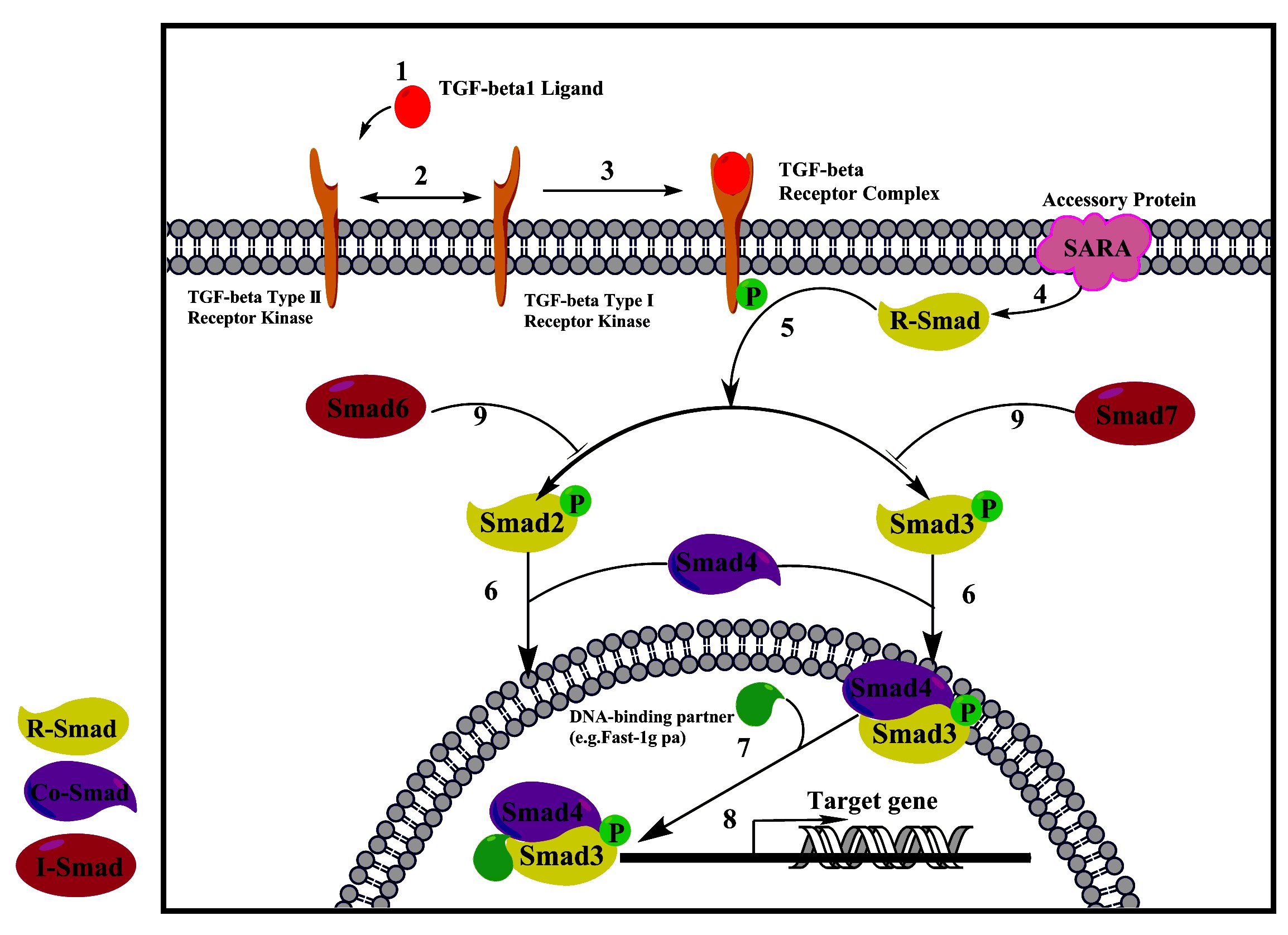


Figure S1: Transforming growth factor-β1 (TGF-β1) docks on a type II (1) and type I TGF-β receptors (TGF-βRI and TGF-β RII) (2). The two receptors then form a receptor complex where TGF-βRI get phosphorylated (3). Subsequently, TGF-βRI phosphorylates the receptor-regulated cytoplasmic proteins (R-Smads) Smad2 and Smad3 (5). This happens with the help of accessory proteins e.g. SARA which is located in the extracellular matrix (ECM) (4). The R-Smads form a complex and bind with the phosphorylated common mediator (co-Smad) Smad4 and transduce into the nucleus (6). There they interact with different DNA proteins, co-activators and co-repressors (7) to induce or suppress the transcription of numerous target genes (8). The inhibitory Smads (I-Smads) Smad6 and Smad7) form a negative feedback and mark the receptors for degradation (9) while R-Smads become inactive by the Smurf effect (modified after [1]).


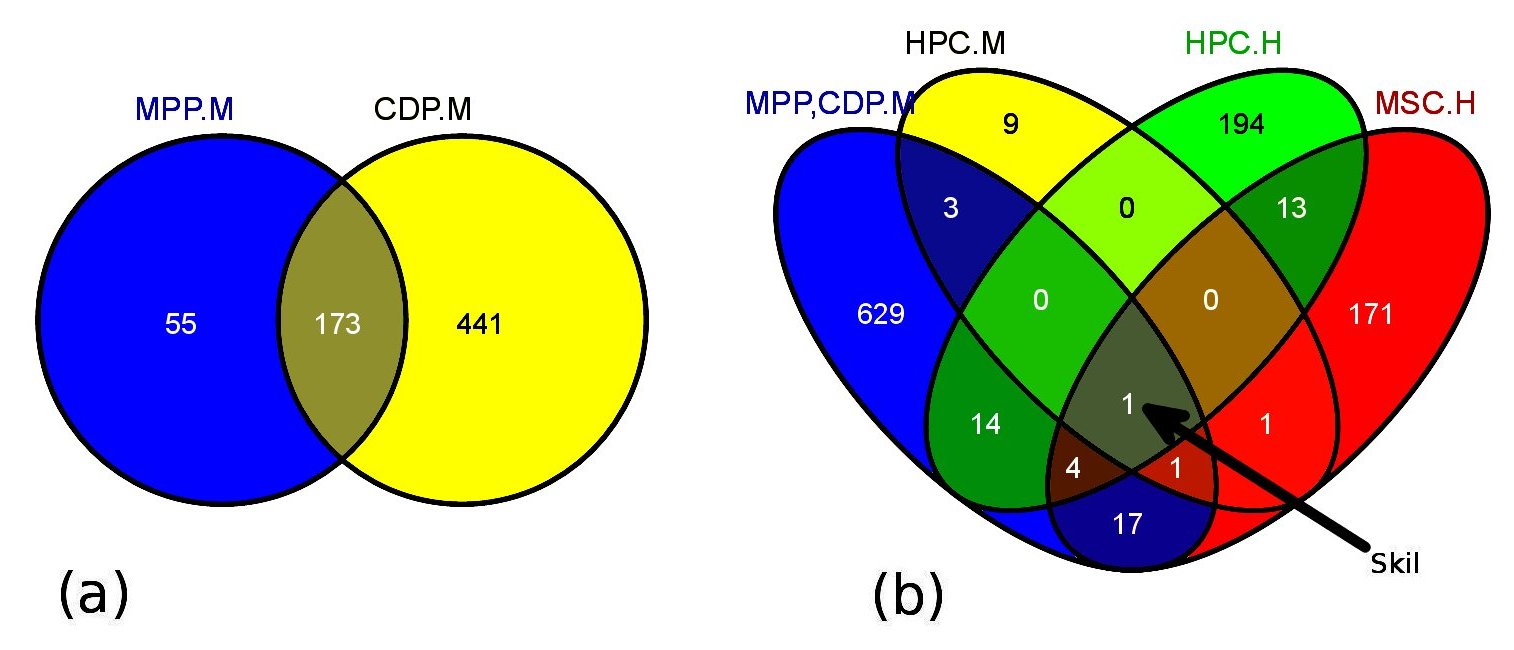


Figure S2: (a) Venn diagram of DE genes (probability ≥ 99% and |logFC| ≥ 1) in mouse MPP and mouse CDP. (b) Union of DE genes in MPP and CDP compared to all other cell types (homolog genes).


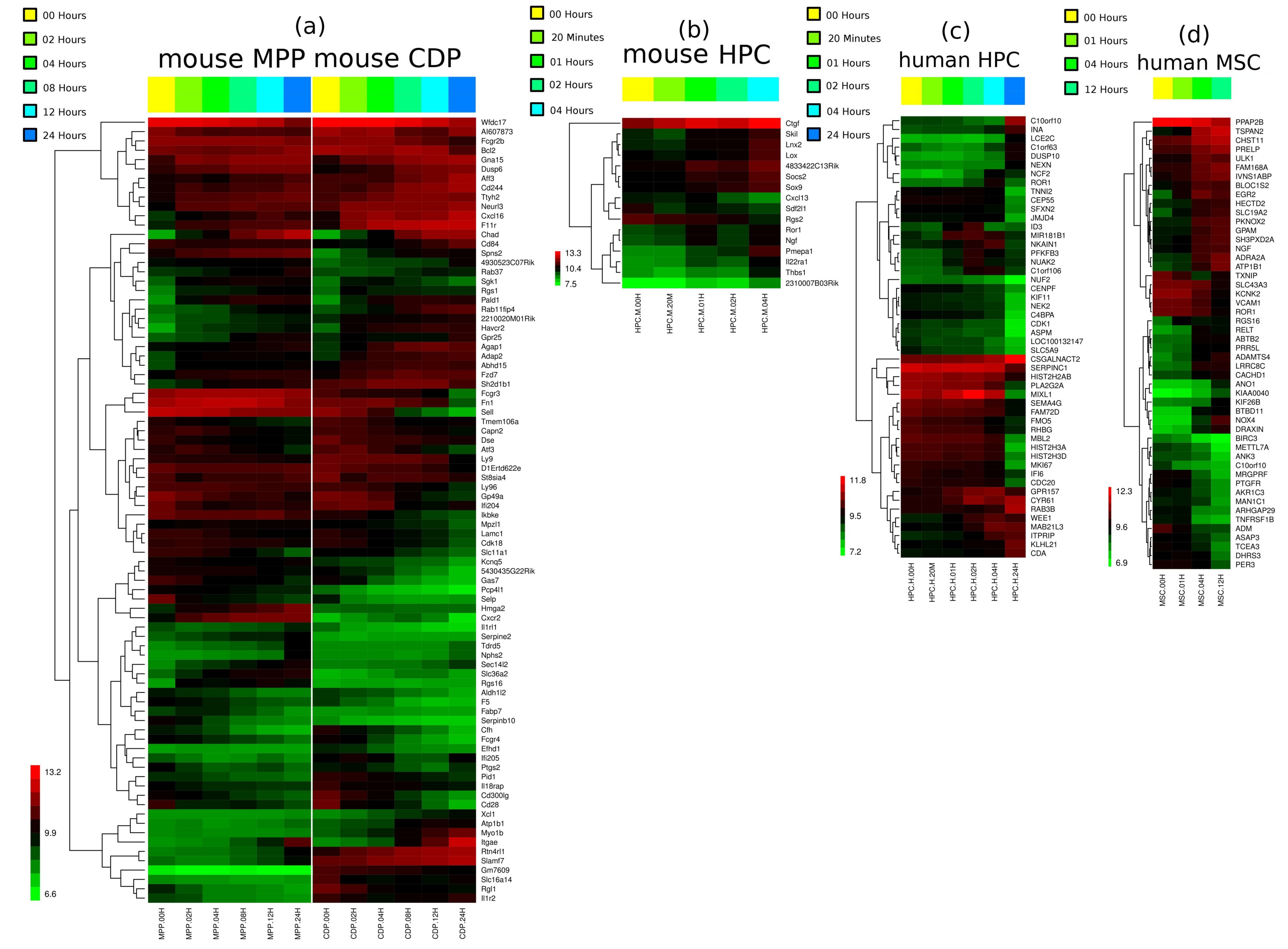


Figure S3: Heatmaps depicting mean log2 fold changes of top DE genes at different time points. (a) Mouse MPP and CDP (together 84 genes), (b) mouse HPC (16 DE genes), (c), (d) top 50 DE genes in HepG2 (HPC) and human MSCs, respectively.


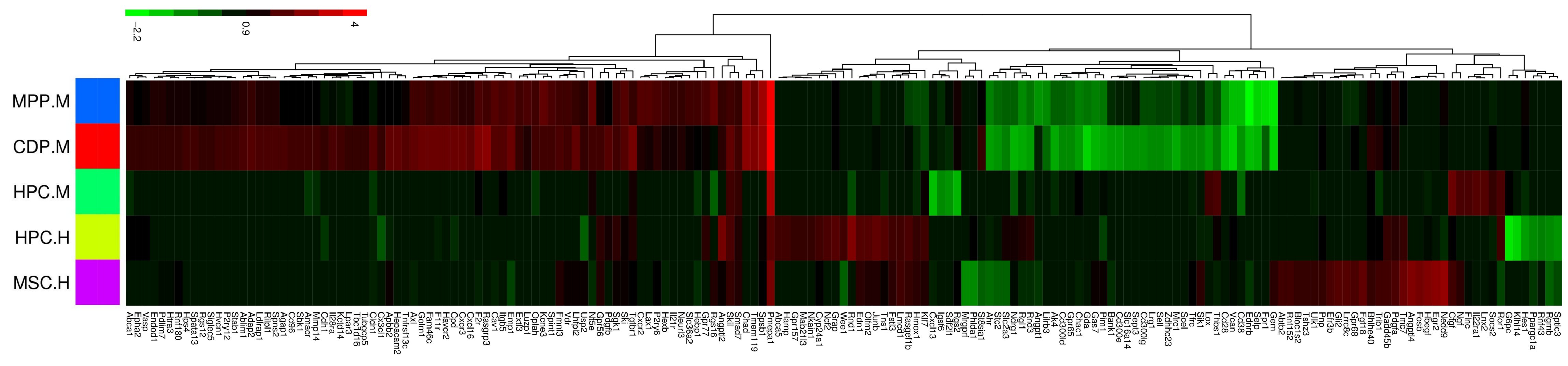


Figure S4: Heatmap depicting log2 fold changes of all genes that are DE genes in at least one cell type 4 hours after stimulation.


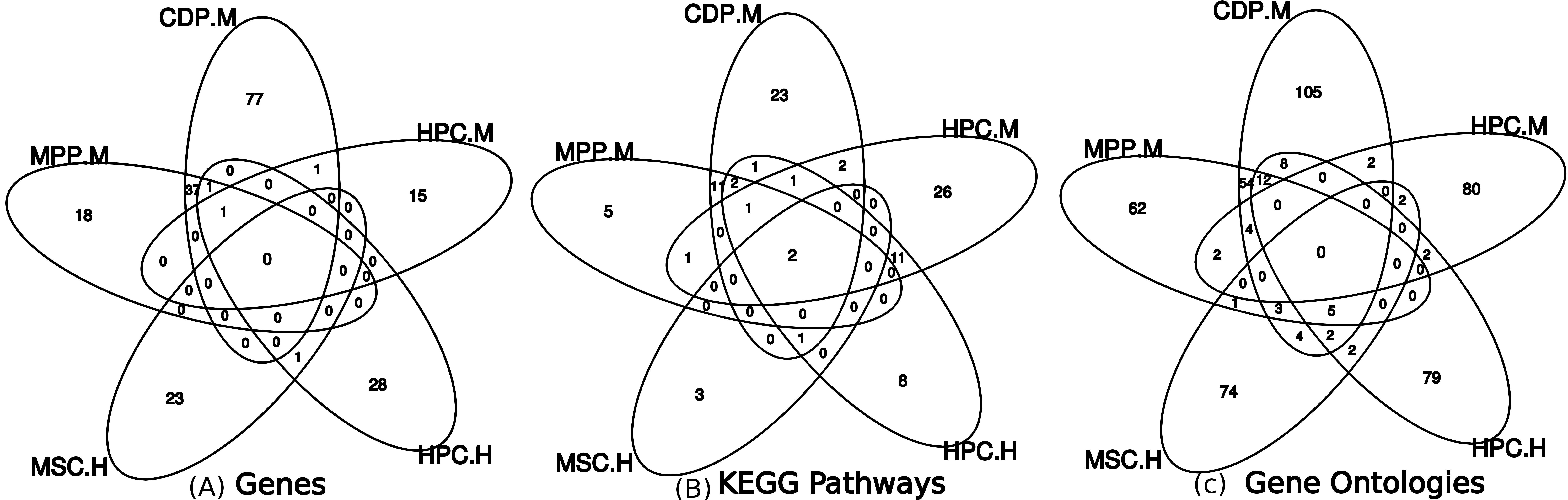


Figure S5: Venn Diagrams of differentially expressed genes (FDR_BH_ < 0.01 and |logFC| >= 1) and associated KEGG pathways and GO terms (FDR_BY_ < 0.05) in mouse MPP, mouse CDP, human HPC and human HPC cell types at time point 4 hours (taking homologous genes between human and mouse into account, as described in section “Material and Methods”, details in the included excel files).


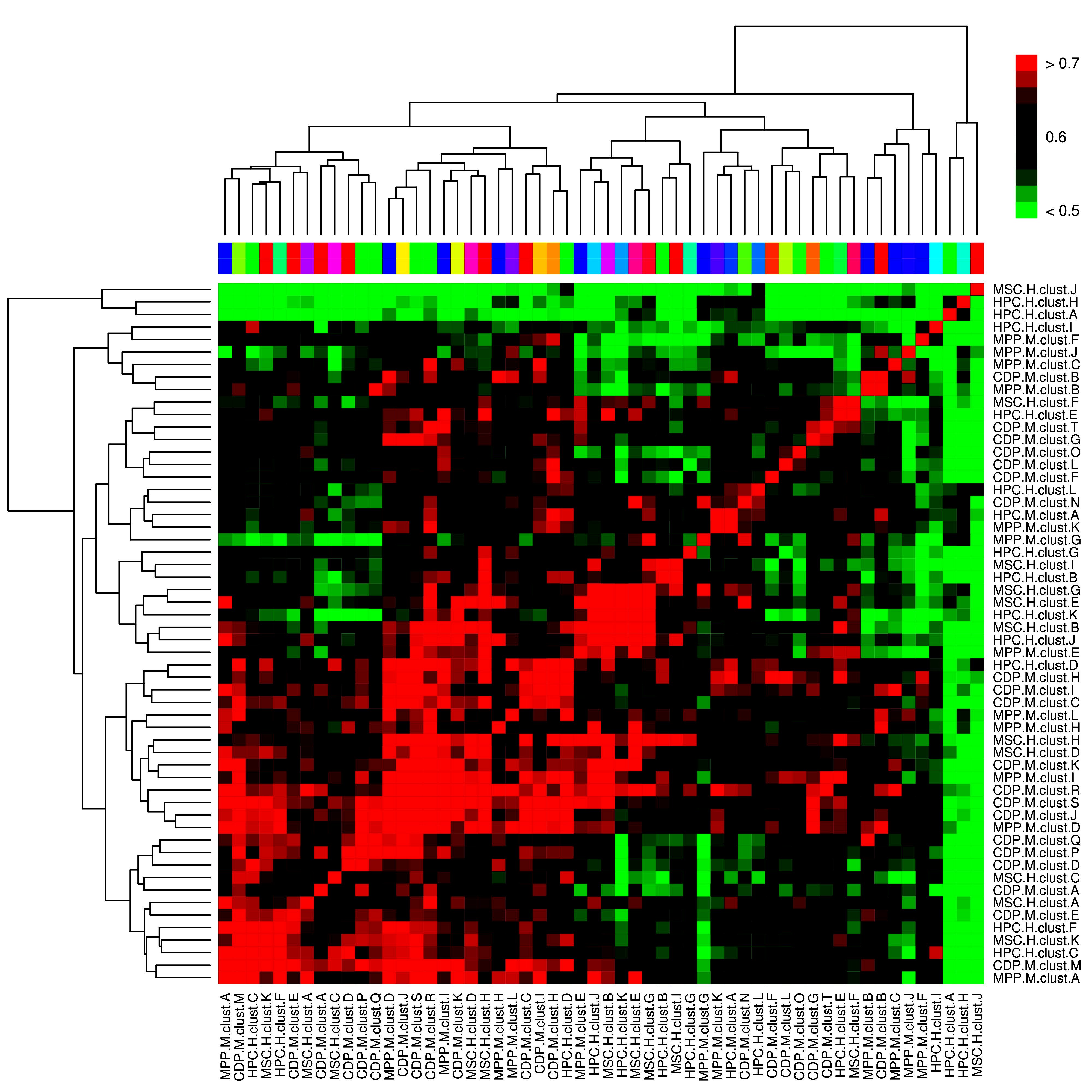


Figure S6: GO semantic similarity heatmap for all the resulting clusters in all cell types. The color code indicates the degree of functional similarity between clusters according to their GO annotation. GO semantic similarities were computed via the Bioconductor R-package “GOSemSim” using the similarity measure by [2].

**Tables:**

Table S1: Overview of the different experiments. Chips for mouse hepatocytes at time point 1h were removed due to quality issues.

| **Organism** | **Mouse** | | | **Human** | |
| --- | --- | --- | --- | --- | --- |
| **Cell Type** | **Multi-potent progenitor (MPP)** | **Comm-Dendritic Progenitor (CDP)** | **Hepatocytes (HPC)** | **Hepatocytes (HPC)** | **Mesenchymal (MSC)** |
| **Replicates** | **3** | **3** | **3** | **3** | **3** |
| **Time Points** | **6** | **6** | **5** | **6** | **4** |
| **00 Hours** | **** | **** | **** | **** | **** |
| **20 Minutes** |  |  | **** | **** |  |
| **01 Hours** |  |  | **** | **** | **** |
| **02 Hours** | **** | **** | **** | **** |  |
| **04 Hours** | **** | **** | **** | **** | **** |
| **08 Hours** | **** | **** |  |  |  |
| **12 Hours** | **** | **** |  |  | **** |
| **24 Hours** | **** | **** |  | **** |  |

Table S2: Number of DE genes (probability ≥ 99% and |logFC| ≥ 1) in each cell type and condition according to the time-course analysis. Comparisons between mouse and human are based on homologous genes (see Material and Methods). The diagonal in the table indicates the number of DE genes in each cell type. The other numbers are pair-wise overlaps.

|  |  | **Mouse** | | | **Human** | |
| --- | --- | --- | --- | --- | --- | --- |
|  |  | **MPP** | **CDP** | **HPC** | **HPC** | **MSC** |
| **Mouse** | **MPP** | **228** | **173** | **2** | **9** | **10** |
|  | **CDP** | **173** | **614** | **4** | **16** | **19** |
|  | **HPC** | **2** | **4** | **15** | **1** | **3** |
| **Human** | **HPC** | **9** | **16** | **1** | **226** | **18** |
|  | **MSC** | **10** | **19** | **3** | **18** | **208** |

Table S3: Differentially expressed genes in each cell type and number of resulting clusters (details in the included excel files table S14).

| Cell Type | MPP.M | CDP.M | HPC.M | HPC.H | MSC.H |
| --- | --- | --- | --- | --- | --- |
| Differentially expressed genes | 230 | 631 | 15 | 232 | 208 |
| # Clusters | 12 | 20 | 1 | 12 | 11 |

Table S4 : Numbers of enriched KEGG pathways (black) and GO terms (red) in each cell type and condition at FDR_BY_ < 0.05 (diagonal) according to time-course analysis. The other numbers are pair-wise overlaps. Underlined numbers are significant overlaps between the corresponding two cell types, according to a hyper-geometric test with P value < 0.05 (details in the included excel files Tables S10 and S11).

| **KEGG** | | **Mouse** | | | **Human** | | |
| --- | --- | --- | --- | --- | --- | --- | --- |
|  |  | **MPP** | **CDP** | **HPC** | **HPC** | **MSC** | **CRL** |
| **Mouse** | **MPP** | **98** | **85** | **22** | **31** | **54** | **57** |
|  | **CDP** | **85** | **116** | **24** | **36** | **58** | **68** |
|  | **HPC** | **22** | **24** | **47** | **16** | **18** | **26** |
| **Human** | **HPC** | **31** | **36** | **16** | **58** | **32** | **37** |
|  | **MSC** | **54** | **58** | **18** | **32** | **84** | **64** |
|  | **CRL** | **57** | **68** | **26** | **37** | **64** | **106** |

| **GO** | | **Mouse** | | | **Human** | | |
| --- | --- | --- | --- | --- | --- | --- | --- |
|  |  | **MPP** | **CDP** | **HPC** | **HPC** | **MSC** | **CRL** |
| **Mouse** | **MPP** | **255** | **166** | **36** | **48** | **68** | **87** |
|  | **CDP** | **166** | **238** | **33** | **55** | **66** | **94** |
|  | **HPC** | **36** | **33** | **139** | **30** | **22** | **35** |
| **Human** | **HPC** | **48** | **55** | **30** | **191** | **73** | **92** |
|  | **MSC** | **68** | **66** | **22** | **73** | **252** | **127** |
|  | **CRL** | **87** | **94** | **35** | **92** | **127** | **504** |

Table S5: Total number of predicted transcription factor binding sites (TFBS) in each cell type and condition (best match according to STAMP and E-value < 1e-3) according to time-course analysis. The other numbers are pair-wise overlaps (details in the included excel files Table S17).

| **TFBS** | | **Mouse** | | | **Human** | |
| --- | --- | --- | --- | --- | --- | --- |
|  |  | **MPP** | **CDP** | **HPC** | **HPC** | **MSC** |
| **Mouse** | **MPP** | **11** | **6** | **0** | **5** | **2** |
|  | **CDP** | **6** | **18** | **0** | **3** | **2** |
|  | **HPC** | **0** | **0** | **0** | **0** | **0** |
| **Human** | **HPC** | **5** | **3** | **0** | **21** | **3** |
|  | **MSC** | **2** | **2** | **0** | **3** | **13** |

Table S6: Significant TFBS in each cell type (best match according to STAMP and E-Value < 1e-03) for the significant genes according to time-course analysis (table S17).

| **MPP.M** |  | **CDP.M** |  | **HPC.M** |  | **HPC.H** |  | **MSC.H** |  |
| --- | --- | --- | --- | --- | --- | --- | --- | --- | --- |
| **TFBS** | **E-value** | **TFBS** | **E-value** | **TFBS** | **E-value** | **TFBS** | **E-value** | **TFBS** | **E-value** |
| SP1SP3_Q4 | 1.11E-16 | KROX_Q6 | 2.32E-13 |  |  | KROX_Q6 | 3.66E-15 | KROX_Q6 | 0.00E+00 |
| FOXP1_01 | 8.93E-14 | PITX2_Q2 | 1.28E-12 |  |  | PITX2_Q2 | 2.61E-12 | SF1_Q6 | 3.24E-11 |
| SP1SP3_Q4 | 3.19E-13 | KROX_Q6 | 3.73E-12 |  |  | TBX5_Q5 | 1.90E-11 | KROX_Q6 | 3.92E-11 |
| KROX_Q6 | 4.48E-11 | FOXP1_01 | 4.61E-12 |  |  | POU1F1_Q6 | 4.56E-11 | PU1_Q6 | 5.49E-09 |
| FOXP1_01 | 2.61E-09 | FOXP1_01 | 8.81E-12 |  |  | HFH4_01 | 3.49E-09 | PIT1_Q6 | 1.75E-08 |
| TEF_Q6 | 1.81E-08 | E2A_Q2 | 2.36E-08 |  |  | SP1SP3_Q4 | 1.74E-08 | POU3F2_01 | 3.90E-08 |
| POU6F1_01 | 1.54E-07 | NFE2_01 | 1.91E-07 |  |  | MAZ_Q6 | 9.64E-08 | PAX4_04 | 1.63E-07 |
| FOX_Q2 | 4.60E-07 | POU3F2_01 | 1.97E-07 |  |  | ZNF219_01 | 1.21E-07 | MYC_Q2 | 6.71E-07 |
| MAZ_Q6 | 1.89E-06 | FOX_Q2 | 2.07E-07 |  |  | RBPJK_Q4 | 1.21E-07 | IRF_Q6 | 1.16E-06 |
| HFH4_01 | 1.06E-05 | POU6F1_01 | 6.61E-07 |  |  | HNF1_Q6_01 | 1.39E-07 | PAX4_04 | 1.89E-05 |
| PITX2_Q2 | 3.10E-05 | TEF_Q6 | 1.19E-06 |  |  | AP2_Q6_01 | 1.76E-07 | HFH4_01 | 3.72E-05 |
|  |  | FOXP1_01 | 2.72E-06 |  |  | TFIII_Q6 | 8.09E-07 | SF1_Q6 | 4.08E-05 |
|  |  | VDR_Q3 | 7.85E-06 |  |  | LFA1_Q6 | 5.72E-06 | FOXM1_01 | 9.98E-04 |
|  |  | YY1_Q6 | 1.01E-05 |  |  | GFI1B_01 | 1.29E-05 |  |  |
|  |  | CACBINDPROTEIN_Q6 | 1.66E-05 |  |  | IRF1_01 | 1.33E-05 |  |  |
|  |  | E2A_Q6 | 6.02E-05 |  |  | PAX4_04 | 2.32E-05 |  |  |
|  |  | E2A_Q2 | 1.25E-04 |  |  | CEBPGAMMA_Q6 | 2.41E-05 |  |  |
|  |  | CACBINDPROTEIN_Q6 | 3.39E-04 |  |  | RREB1_01 | 5.67E-05 |  |  |
|  |  |  |  |  |  | AHR_01 | 6.62E-05 |  |  |
|  |  |  |  |  |  | E2A_Q2 | 1.69E-04 |  |  |
|  |  |  |  |  |  | LFA1_Q6 | 1.81E-04 |  |  |

Table S7: Significant TFBS in each cell type (best match according to STAMP and E-Value < 1e-03) for the significant genes at time point 4 hours (Tables S18).

| **MPP.M** |  | **CDP.M** |  | **HPC.M** |  | **HPC.H** |  | **MSC.H** |  |
| --- | --- | --- | --- | --- | --- | --- | --- | --- | --- |
| **TFBS** | **E-value** | **TFBS** | **E-value** | **TFBS** | **E-value** | **TFBS** | **E-value** | **TFBS** | **E-value** |
| FOXP1_01 | 1.11E-16 | FOXP1_01 | 8.91E-10 | ZF5_01 | 5.91E-08 | KROX_Q6 | 0.00E+00 | CKROX_Q2 | 1.23E-10 |
| SP1SP3_Q4 | 3.00E-15 | PIT1_Q6 | 4.08E-08 | HFH4_01 | 1.00E-05 | SP1SP3_Q4 | 1.60E-10 | PIT1_Q6 | 2.94E-10 |
| ZF5_01 | 8.69E-11 | HEN1_01 | 1.34E-07 |  |  | AP2_Q6 | 2.26E-07 | HNF1_Q6 | 6.46E-10 |
| SP1_Q6_01 | 6.17E-10 | DMRT5_01 | 1.68E-07 |  |  | POU1F1_Q6 | 8.11E-06 | KROX_Q6 | 4.57E-09 |
| TEF_Q6 | 9.35E-07 | TEF_Q6 | 1.73E-06 |  |  | HFH4_01 | 2.36E-05 | KROX_Q6 | 1.18E-07 |
| AP2_Q6 | 3.25E-06 | AP2_Q6_01 | 3.07E-06 |  |  | SF1_Q6 | 1.03E-04 | PAX4_04 | 1.79E-07 |
| PPARG_02 | 2.50E-04 | E2F_Q2 | 7.81E-05 |  |  | CDC5_01 | 2.00E-04 | ZNF219_01 | 7.99E-07 |
|  |  |  |  |  |  |  |  | TFE_Q6 | 2.09E-05 |

**Further Tables (in excel format):**

**Table S8: Time-course differential expression analyses results of all cell types.**

**Table S9: Time-point differential expression analyses at 04 hours results of all cell types.**

**Table S10: Time-course KEGG pathways analyses results for all cell types.**

**Table S11: Time-course Gene ontology analyses results for all cell types.**

**Table S12: KEGG pathways analyses at 4 hours for all cell types.**

**Table S13: Gene ontology analyses at 4 hours for all cell types.**

**Table S14: Cluster Analyses: gene assignment lists.**

**Table S15: Gene-set enrichment analyses of KEGG pathways in cluster groups.**

**Table S16: Gene-set enrichment analyses of gene ontology in cluster groups.**

**Table S17: Transcription factors binding sites analyses of differential time-course genes.**

**Table S18: Transcription factors binding site analyses of differential genes at 4 hours.**

**References:**

1. Massagué J: **TGF-β signal transduction**. *Annual Review of Biochemistry* 1998, **67**:753–791.

2. Wang JZ, Du Z, Payattakool R, Yu PS, Chen C-F: **A new method to measure the semantic similarity of GO terms.** *Bioinformatics* 2007, **23**:1274–1281.
